# Interaction and Regulation of Carbon, Nitrogen, and Phosphorus Metabolisms in Root Nodules of Legumes

**DOI:** 10.3389/fpls.2018.01860

**Published:** 2018-12-18

**Authors:** Ailin Liu, Carolina A. Contador, Kejing Fan, Hon-Ming Lam

**Affiliations:** ^1^Centre for Soybean Research, State Key Laboratory of Agrobiotechnology, Shatin, Hong Kong; ^2^School of Life Sciences, The Chinese University of Hong Kong, Shatin, Hong Kong

**Keywords:** rhizobia, bacteroids, nitrogen fixation, legumes, root nodule, phosphate homeostasis, metabolism, symbiosis

## Abstract

Members of the plant family Leguminosae (Fabaceae) are unique in that they have evolved a symbiotic relationship with rhizobia (a group of soil bacteria that can fix atmospheric nitrogen). Rhizobia infect and form root nodules on their specific host plants before differentiating into bacteroids, the symbiotic form of rhizobia. This complex relationship involves the supply of C_4_-dicarboxylate and phosphate by the host plants to the microsymbionts that utilize them in the energy-intensive process of fixing atmospheric nitrogen into ammonium, which is in turn made available to the host plants as a source of nitrogen, a macronutrient for growth. Although nitrogen-fixing bacteroids are no longer growing, they are metabolically active. The symbiotic process is complex and tightly regulated by both the host plants and the bacteroids. The metabolic pathways of carbon, nitrogen, and phosphate are heavily regulated in the host plants, as they need to strike a fine balance between satisfying their own needs as well as those of the microsymbionts. A network of transporters for the various metabolites are responsible for the trafficking of these essential molecules between the two partners through the symbiosome membrane (plant-derived membrane surrounding the bacteroid), and these are in turn regulated by various transcription factors that control their expressions under different environmental conditions. Understanding this complex process of symbiotic nitrogen fixation is vital in promoting sustainable agriculture and enhancing soil fertility.

## Introduction

Leguminosae (Fabaceae) is the third largest family of angiosperms with 750 genera and around 19,500 species (The Legume Phylogeny Working Group, 2013). Most legumes can establish a mutualistic association with alpha- and beta-proteobacteria to obtain biological nitrogen (reviewed by Andrews and Andrews, 2017; Sprent et al., 2017). Rhizobia are soil bacteria known for being able to establish symbiosis with legume plants. Symbiotic nitrogen fixation (SNF) can be carried out once rhizobia are established inside the cells of root nodules formed from newly differentiated tissue in the roots of host plants. The host plant provides the microsymbiont with dicarboxylates together with other nutrients, in exchange for fixed nitrogen in the form of ammonium and amino acids (Udvardi and Day, 1997). Nitrogen-fixing legumes contribute to nitrogen enrichment of the soil and therefore are valuable in improving soil fertility. The legume–rhizobium association has an important impact on sustainable agriculture since it provides more than 65% of the biologically fixed nitrogen in agricultural systems (Herridge et al., 2008).

Studies on legume–rhizobium symbiosis have covered a large number of legume species, such as soybean (*Glycine max*), bird’s foot trefoil (*Lotus japonicus*), alfalfa (*Medicago sativa*), barrelclover (*Medicago truncatula*), common bean (*Phaseolus vulgaris*), garden pea (*Pisum sativum*), common vetch (*Vicia sativa*), and narrowleaf lupin (*Lupinus angustifolius*) (Dupont et al., 2012). Although *M. sativa*–*Sinorhizobium meliloti* and *P. sativum*–*Rhizobium leguminosarum* associations are also well-studied symbiotic system (Kneen and LaRue, 1984; Jones et al., 2007), the genetic models of legume–rhizobium symbiosis are mainly focused on *L. japonicus–Mesorhizobium loti* and *M. truncatula–S. meliloti* associations, due to the small diploid genomes, high levels of genetic diversity, and well-established transformation systems of *L. japonicus* and *M. truncatula* (Oldroyd and Geurts, 2001; Oldroyd, 2005). *L. japonicus* possesses determinate root nodules, which are spherical with a well-defined, homogeneous central fixation zone composed of infected rhizobia-filled cells surrounded by uninfected cells (Schultze and Kondorosi, 1998). In contrast, *M. truncatula* forms indeterminate nodules which are cylindrical and consist of a gradient of developmental zones (Timmers et al., 1999).

Owing to their complex makeup, legume nodules have been extensively studied with respect to their metabolism and regulation. After the SNF process has been established in mature nodules, several biological processes occur simultaneously inside the nodules, including biological nitrogen fixation carried out by bacteroids, carbon–nitrogen metabolism, and exchange between host plants and bacteroids, and metabolite transport across cell membranes (Resendis-Antonio et al., 2011; Udvardi and Poole, 2013; Clarke et al., 2014). Root nodules mediate the influx of carbon sources and efflux of nitrogen compounds. The host plant supplies sucrose which will be converted to sources of energy and organic acids for fixation of atmospheric nitrogen, while the endosymbiont returns organic fixation products to the host (Day et al., 2001). Moreover, SNF in legume nodules exerts a high demand for phosphates (Gunawardena et al., 1992; Saad and Lam-Son, 2017). The phosphate concentration in nodules is threefold higher than in other organs or tissues (Sa and Israel, 1991). Under phosphate starvation, the phosphate acquisition rate increases in nodules to make them less vulnerable compared to other organs (Thuynsma et al., 2014; Saad and Lam-Son, 2017). Optimal plant phosphate requirement is about 0.05–0.30% of total dry weight, and inorganic phosphate is the main form absorbed by plants (Temple et al., 1998; Saad and Lam-Son, 2017).

Better understanding of the metabolic components participating in N_2_ fixation and the key regulators that control the processes in legume nodules are a crucial step in seeking possible ways to enhance SNF and further increase legume productivity. In this review, we focus on the latest knowledge on root nodules including the nodule types (the section “Legume Nodule Types and Their Compatible Rhizobia”), the metabolic changes, transportation and regulation mechanisms in host plant (the section “Overview of Metabolism and Regulation in the Host Plant”) and bacteroids (the section “Metabolism and Transport in Bacteroids”).

## Legume Nodule Types and their Compatible Rhizobia

About 90% of the species within the family Leguminosae (Fabaceae) can fix atmospheric nitrogen through a symbiotic association with soil bacteria known as rhizobia (Rascio and La Rocca, 2013; Andrews and Andrews, 2017). Rhizobia are diazotrophic Gram-negative bacteria able to form nitrogen-fixing nodules on the roots of legumes, where they differentiate into bacteroids (Rosenberg et al., 2014). Rhizobia present different specificities toward different host plant species based on the recognition of specific signal molecules (Nod factors) (Spaink, 2000; Poole et al., 2018). However, promiscuity has been observed in some cases. For example, *Sinorhizobium fredii* strain NGR234 is able to establish symbiosis with over 100 plant legumes such as cultivated soybean (*G. max*), wild soybean (*Glycine soja*), pigeon pea (*Cajanus cajan*), and cowpea (*Vigna unguiculata*) (reviewed in Brenner et al., 2009). Recently, analysis on legume–rhizobia symbioses was published where specificity (reviewed in Andrews and Andrews, 2017) and biogeography distribution (reviewed in Sprent et al., 2017) of nodulating legumes and their symbionts were discussed in detail.

Nodules are divided into two types: determinate and indeterminate. Determinate nodules are characteristic of soybean and common bean. Legumes such as alfalfa and garden pea present indeterminate nodules. Indeterminate nodules display five developmental zones: meristem (Zone I), infection and differentiation (Zone II), transition region between Zone II and Zone III in which bacteria are engulfed by plant cells (interzone II–III), nitrogen fixation (Zone III), and senescence (Zone IV) (Vasse et al., 1990). The meristem is active in indeterminate nodules and continues to grow, unlike in determinate nodules where meristem cells die once the nodules are mature. The meristem is the distal zone to the root and Zone IV is the proximal to the root attachment site. Zone-specific transcriptional and metabolic changes have been reported in the nodules formed between *M. truncatula* with *S. meliloti* and *S. medicae*, respectively (Roux et al., 2014; Ogden et al., 2017). A fifth zone (Zone V) has been described in alfalfa nodules (Timmers et al., 2000). This zone is proximal to the senescence zone and contains saprophytic intracellular rhizobia that do not undergo bacteroid differentiation. Table 1 summarizes examples of legume–rhizobium associations and nodule types.

**Table 1:** Rhizobia, host plant associations, and nodule types.

Nodule type	Rhizobia	Host plant
Determinate	*Mesorhizobium loti*	*Lotus japonicum*
	*Rhizobium tropici*	*Phaseolus vulgaris*
	*Bradyrhizobium japonicum*	*Glycine max*
		*Vigna unguiculata*
	*Sinorhizobium fredii*	*Glycine max*
		*Vigna unguiculate*
		*Cajanus cajan*
		*Glycine soja*
	*Rhizobium leguminosarum bv. trifolii*	*Phaseolus vulgaris*
		*Trifolium repens*
	*Rhizobium etli*	*Phaseolus vulgaris*
	*Rhizobium leguminosarum bv. phaseoli*	*Phaseolus vulgaris*
	*Bradyrhizobium* spp.	*Aeschynomene afraspera*
		*Aeschynomene indica*
Indeterminate	*Rhizobium leguminosarum bv. viciae*	*Pisum sativum*
		*Vicia cracca*
		*Vicia hirsute*
		*Vicia faba*
		*Lens culinaris Medik*
	*Sinorhizobium meliloti*	*Medicago sativa*
		*Medicago truncatula*
	*Mesorhizobium loti*	*Leucaena leucocephala*
	*Mesorhizobium cicero*	*Cicer arietinum*
	*Mesorhizobium mediterranum*	*Cicer arietinum*
	*Cupriavidus taiwanensis*	*Mimosa pudica*
		*Mimosa diplotricha*
		*Mimosa priga*
	*Burkholderia* spp.	*Mimosa pudica*
		*Mimosa priga*
Determinate and indeterminate	*Azorhizobium caulinodans*	*Sesbania rostrata*

## Overview of Metabolism and Regulation in the Host Plant

### Carbon Metabolism

**FIGURE 1:**
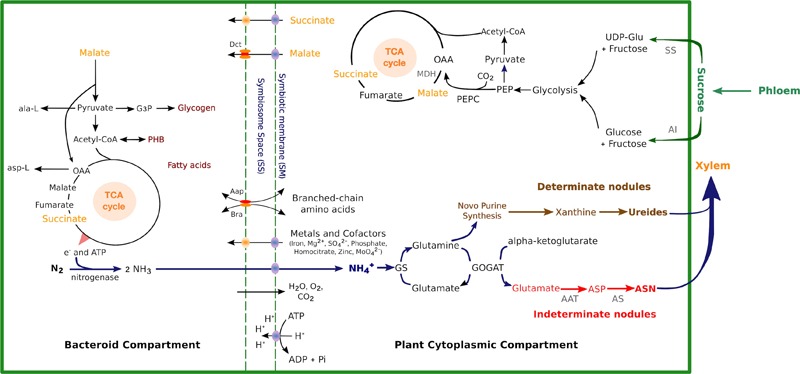
Schematics of carbon and nitrogen metabolic pathways with key enzymes, metabolites, and transporters in determinate nodules and indeterminate nodules. Sucrose in the plant cytosol is split into glucose and fructose by AI or UDP-Glc and fructose via SS, which is then catabolized via glycolysis to PEP. Carbon from PEP and carbonic acid is diverted to OAA and then malate by PEPC and the neMDH, respectively. OAA may be further converted to succinate or fumarate. Carbon sources are transported across the peribacteroid and bacteroid membranes and enter the TCA cycle in the bacteroid to be metabolized. Transport of inorganic ions and cofactors required for SNF across the SM is indicated. The ammonia produced by the SNF is transported back to the plant and assimilated by GS and GOGAT into Gln and Glu (blue arrows). In indeterminate nodules, Glu and Gln are further converted to Asp and Asn by AAT and AS, respectively (red arrows). In determinate nodules, Gln enters purine synthesis pathway and is converted to ureides (brown arrows). AI, alkaline invertase; UDP-Glc, UDP-glucose; SS, sucrose synthase; PEP, phosphoenolpyruvate; OAA, oxaloacetate; PEPC, PEP-carboxylase; MDH, malate dehydrogenase; PHB, polyhydroxybutyrate; AAT, aspartate aminotransferase; AS, asparagine synthetase; ASP, aspartate; ASN, asparagine.

Sucrose is the primary carbon resource for energy supply and carbon skeletons for SNF and is supplied to legume nodules by transportation from the shoots (Gordon et al., 1998). However, sucrose is not used directly as a substrate by isolated nitrogen-fixing bacteroids to support nitrogenase activity, as demonstrated in the cases of soybean (Stovall and Cole, 1978), garden pea (Glenn and Dilworth, 1981), lupin (Tomaszewska et al., 1991), and alfalfa (Miller et al., 1988). In nodules, sucrose can be processed by one of two enzymes, sucrose synthase (SS; EC 2.4.1.13) and alkaline invertase (AI; EC 3.2.1.26), as shown in Figure 1. SS performs a reversible UDP-dependent cleavage of sucrose into UDP-glucose (UDP-Glc) and fructose (Akazawa and Okamoto, 1980). SS activity is reduced in the SNF-defective mutant, *rug4*, of *P. sativum*, indicating that SS plays an important role in nodule functions (Gordon et al., 1998, 1999). Moreover, overexpressing the antisense of *MtSucS1* (encoding enzyme SS) in *M. truncatula* impaired the plant growth and nodulation in the *MtSucS1*-reduced nodules, and the contents of amino acids and their derivatives were also found to be reduced, which suggest SS is a crucial player in the establishment and maintenance of an efficient SNF process (Baier et al., 2007). Six SS isoforms have been identified, and the mutants of SS isoform *sus1-1* and *sus3-1* showed 38% and 67% reduction of SS activity, respectively, in nodules formed by inoculating *L. japonicus* with *M. loti* strain Tono, while the double mutant plants of *sus1-1/sus3-1* had significantly impaired growth and their nodule SS activities were reduced by 94% compared to the wild type under the same growth conditions. This further shows that SS is essential in nodulation maintenance (Horst et al., 2007). In addition, when exposed to drought stress, nitrogen fixation in nodules declined, and the activity of SS was severely inhibited in various species (*M. sativa, P. sativum*, and *P. vulgaris*), and led to the further limitation of carbon availability in bacteroids (Ramos et al., 1999; Gálvez et al., 2005; Naya et al., 2007).

The other sucrose-catabolizing enzyme, AI, is a hydrolase, first identified in soybean nodules, that irreversibly cleaves sucrose into fructose and glucose (Copeland and Morell, 1985). The isolation and purification of AI was also carried out from chickpea nodules (Asthir and Singh, 1997). In *L. japonicus*, the expression level of gene *LjInv1* encoding enzyme AI was twofold higher in mature nodules than in uninfected roots. Enzyme activity assays showed that LjInv1 contributed to the production of hexoses and other biosynthetic processes in developing nodules (Flemetakis et al., 2006). In *G. max* and *M. truncatula* root nodules, an alkaline/neutral *Inv* gene was found to have enhanced transcription levels in developing root nodules inferring an increased need for sucrose degradation (Flemetakis et al., 2006; Tesfaye et al., 2006). However, there is not much direct evidence to prove that AI is essential for the regulation of nodule metabolism.

The resulting UDP-Glc and free hexoses (glucose and fructose) are phosphorylated by hexokinase (EC 2.7.1.1) and enter glycolysis or oxidative pentose phosphate pathways. In nodules, the host plant provides carbon sources for bacteroid activities in the form of dicarboxylates, particularly malate and succinate (Day, 1991). Phosphoenolpyruvate carboxylase (PEPC; EC 4.1.1.31) and malate dehydrogenase (MDH; EC 1.1.1.82) convert the carbon flux from glycolysis to form malate (Figure 1). PEPC catalyzes the conversion of PEP into oxaloacetate (OAA). The active enzymes are less sensitive to malate, which act as a negative feedback control (Nimmo, 2000). The nodule PEPC was found to be activated with phosphorylation and its sensitivity was inhibited by L-malate *in vitro* and *in vivo* (Schuller and Werner, 1993; Zhang et al., 1995). In *L. japonicus*, abundant transcript of *LjPEPC1* was found in the vascular bundles of nodules and bacteroid-infected cells, while *LjPEPC2* was expressed in roots and shoots at low levels and postulated as the housekeeping isoform. Moreover, regulatory phosphorylation of PEPC is thought to be mainly controlled by the PEPC kinase (PEPC-PK) (Vidal and Chollet, 1997), and the expression of *LjPEPC-PK* shares a similar pattern with *LjPEPC1* in mature nodules (Nakagawa et al., 2003). The OAA is then converted to malate by MDH in a reversible reaction, and this can then be uptaken by bacteroids directly (Figure 1). In alfalfa, five species of cDNAs were found to encode MDH, but only one of them had a nodule-enhanced expression pattern (Miller et al., 1998). One nodule-enhanced MDH (neMDH) was found in garden pea (Fedorova M. et al., 1999). Two MDH isoforms were found in the *L. japonicus* nodule by transcriptome profiling, one of which is a cytosolic MDH (cMDH) and the other a neMDH (Takanashi et al., 2012). The activity of cMDH in *L. angustifolius* nodules was shown to increase under P-deficient conditions, supporting the involvement of cMDH in nodules (Le Roux et al., 2014).

Besides the enzymes described above, some other carbon metabolic enzymes also play a role in legume nodules. *MsSPSA*, encoding sucrose phosphate synthase (SPS; EC 2.3.1.14) in *M. sativa* L., is involved in the first step of sucrose synthesis, and the expression of *MsSPSA* showed a nodule-enhanced pattern. SPS showed higher activities in the nodules formed by wild-type *S. meliloti*, compared to nodules inoculated with a N-deficient Fix^-^ strain (a Tn5 insertional mutation in *nifH*; Hirsch et al., 1983), suggesting an important role in maintaining a stable carbohydrate/energy supply for the actively nitrogen-fixing symbiont (Aleman et al., 2010).

Multiple environmental stresses result in the inhibition of carbon metabolism in host legumes, resulting in the suppression of SNF efficiency in nodules. Under salt stress, the concentration of sucrose was decreased, and major carbon metabolic enzymes such as SS and AI showed lower activity in both *M. truncatula* nodules (indeterminate nodules) and *L. japonicus* nodules (determinate nodules) (López et al., 2008). When garden pea plants were cultured under drought stress, the SS activity was down-regulated, indicating the normal carbon metabolic pathway was inhibited. The NADP-dependent isocitrate dehydrogenase (ICDH; EC1.1.1.42) was reported to balance C/N metabolic fluxes in leaves (Gallardo et al., 1995; Gálvez and Gadal, 1995). The enhancement of ICDH activity in nodules was probably the result of compensating for the C and N imbalance or supplying additional NADPH to neutralize the effects of the increased production of reactive oxygen species (ROS) as a consequence of drought stress (Gálvez et al., 2005).

### Nitrogen Metabolism

Ammonia exported from the bacteroids diffuses into the cytosol of the infected host cells to be rapidly assimilated. Amino acids and/or ureides are then synthesized and exported from the legume nodules to the shoots (Figure 1). The determinate-nodule legumes primarily transport allantoin and allantoate (ureides) as fixed-N compounds, while the indeterminate nodules assimilate amides in the form of asparagine (Asn) and glutamine (Gln) (Sprent, 2009). The first step of ammonium assimilation is apparently the same in amide- and ureide-exporting legume nodules (Todd et al., 2006). In both cases, nitrogen fixed in the bacteroids is exported into the host cell cytosol, where ammonia is converted to Gln and glutamate (Glu) by the enzymes Gln synthetase (GS; EC 6.3.1.2) and Glu synthase (NADH-GOGAT; EC 1.4.1.14). In indeterminate nodules, Glu and Gln are further converted to aspartate (Asp) and Asn by Asp aminotransferase (AAT EC 2.6.1.1) and Asn synthetase (AS; EC 6.3.5.4). While for determinate nodules, Gln further entry purine synthesis pathway and convert to ureides as final product exported in to host plant (Figure 1).

Glutamine synthetase, as the enzyme for the first step of ammonia assimilation in the infected root cells, is a key component of nitrogen metabolism in nodules. It catalyzes the ATP-dependent amination of Glu to Gln. GS possesses two major isoforms largely separated by their subcellular localization, plastidic GS2 and cytosolic GS1 (Temple et al., 1998; Lea and Miflin, 2003). In *M. truncatula* nodules, three *GS* genes have been identified: cytosolic *MtGS1a* and *MtGS1b*, and plastid *MtGS2*, where MtGS1a accounts for 90% of the GS activity in nodules (Carvalho et al., 1997, 2000). Impairment of GS activity using phosphinothricin resulted in the inhibition of nodule growth, and the promotion of nodule senescence. The reduced activity of GS also suppressed Asn biosynthesis, as demonstrated by the lower expression of *AS* transcripts and lower Asn content (Seabra et al., 2012). NADPH-GOGAT is another enzyme involved in the assimilation of ${\mathrm{NH}}_{4}^{+}$, by transferring the amide group from Gln to α-ketoglutarate (Figure 1). In transgenic *M. sativa* expressing antisense *NADPH-GOGAT*, GOGAT activities were much reduced in the nodules. The transgenic *M. sativa* inoculated with *S. meliloti* exhibited moderate chlorosis, lower nodule weight, and reduction of N_2_ fixation efficiency compared to the control (Schoenbeck et al., 2000).

In addition to primary nitrogen assimilation by converting ammonia into amino acids, ureide biosynthesis is also a common pathway among tropical legumes such as soybean, common bean, and cowpea, which generally form determinate nodules (Tajima, 2004). The allantoin and allantoate are the final nitrogen forms exported from soybean nodules to the shoots (McClure and Israel, 1979). Before ureide synthesis, ammonia enters a purine synthesis pathway, which is catalyzed by xanthine oxidase and xanthine dehydrogenase into urate, whose activities have been detected in both infected cells and uninfected cells in cowpea nodules. While uricase, which catalyzes the irreversible conversion of urate to allantoin, was detected in uninfected cells but not infected cells (Atkins et al., 1997). Moreover, the genes *GmALN3* and *GmALN4*, encoding the enzyme allantoinase (EC 3.5.2.5) that converts allantoin to allantoate, were expressed in soybean nodules (Duran and Todd, 2012). Gln may also be enzymatically transformed into xanthine through the *de novo* purine synthesis pathway (Werner and Witte, 2011; Figure 1).

### Phosphate Metabolism

Phosphate is the second most limiting macronutrient element required for crop growth after nitrogen, and there is a particularly high demand for phosphates in the nodules of N_2_-fixing legumes (Gunawardena et al., 1992; Vance et al., 2000). Phosphate, as an essential macronutrient, is incorporated into organic compounds such as nucleic acids (DNAs and RNAs), enzymes, ATP, sugar phosphates, phospholipids, and so on. Such organic phosphates are involved in many plant biochemical processes such as nutrient transport and photosynthesis. As a component of chromosomes, phosphates play an important role in cell division and organogenesis, and help to transfer the genetic information from one generation to the next (Ahemad and Oves, 2011). The soluble inorganic phosphate (Pi) is the most abundant metabolite in *Bradyrhizobium japonicum* cells. The Pi concentration in free-living *B. japonicum* cells is 8.1 μmol g^-1^ FW and 29.2 μmol g^-1^ FW in bacteroids. Massive amounts of Pi are transported into bacteroids through phosphate transporters and are highly involved in the metabolism of bacteroids. Indeed, SNF expends a great quantity of energy involving phosphorylated intermediates (Vauclare et al., 2013).

#### Legume Metabolic Changes Under Pi Deficiency

Deprivation of Pi can lead to an ATP shortage and a decrease in key metabolic enzymes (e.g., those involved in photosynthesis), and therefore is tightly correlated with plant growth (Czarnecki et al., 2013; Saad and Lam-Son, 2017). Although low Pi availability causes a decline in SNF, the responses to Pi deficiency vary among different legumes. There are two main strategies: one is to make changes to nitrogen and carbon metabolism, such as N acquisition and assimilation as well as carbon source adjustments, and the other is to enhance Pi uptake and recycling in nodules (Wang et al., 2010; Qin et al., 2012; Udvardi and Poole, 2013).

To investigate the metabolic changes in legume nodules under low-Pi stress, metabolic profiling has been done in common bean and chickpea (Hernandez et al., 2009; Zhang N. et al., 2012). Hernandez et al. (2009) performed a metabolic profiling using common bean plants inoculated with *Rhizobium tropici* CIA899 cultivated under Pi-deficient and Pi-sufficient conditions. The levels of 13 metabolites were significantly changed when comparing between the two conditions. Among these, amino acids and other nitrogen metabolites, such as urea, spermidine, and putrescine, were decreased in nodules under low-Pi conditions while most of the carbon metabolites including organic acids and polyhydroxy acids were increased (Hernandez et al., 2009). Similar changes were also found in chickpea inoculated with two different *Mesorhizobium* strains, *M. ciceri* CP-31 (*Mc*CP-31) and *M. mediterranum* SWRI9 (*Mm*SWRI9) (Nasr Esfahani et al., 2016). These two *Mesorhizobium* strains inoculated on chickpea showed a differential symbiotic performance under both control and low-Pi condition. *Mc*Cp-31-chickpea exhibited a higher symbiotic efficiency and higher Pi concentration than *Mm*SWRI9–chickpea under normal and low-Pi conditions. The differences in SNF efficiency between the two types of chickpea–*Mesorhizobium* associations were correlated with various changes in carbon and nitrogen metabolites. For instance, the activities of most carbon and nitrogen metabolic enzymes, such as AAT, GS, and MDH, were increased in *Mc*Cp-31–chickpea nodules, while the activities of these same enzymes were either the same or reduced in *Mm*SWRI9–chickpea nodules. These results provide evidence that host plants adjust their carbon and nitrogen metabolic pathway to maintain the SNF efficiency under Pi deficiency condition (Nasr Esfahani et al., 2016).

**FIGURE 2:**
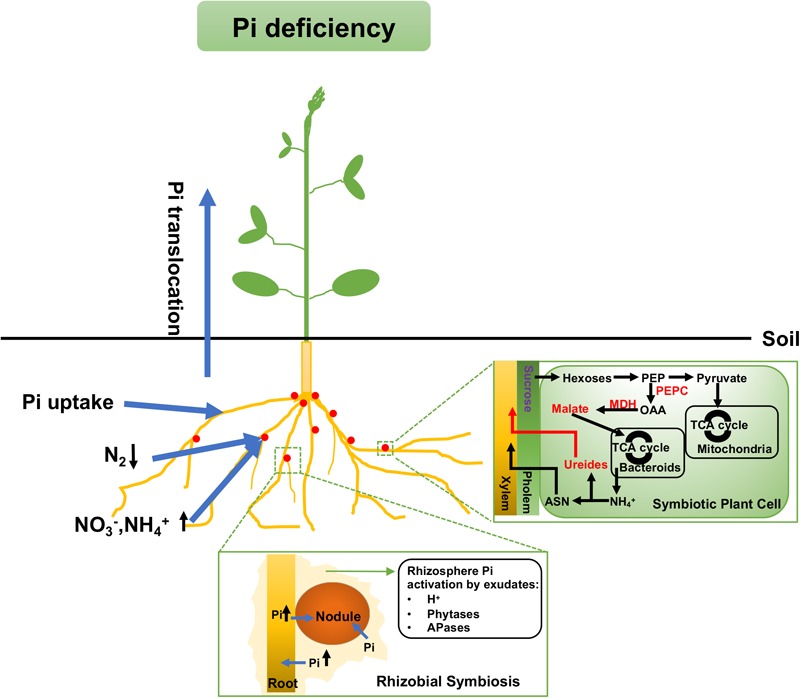
Mechanisms for maintaining Pi homeostasis under Pi deficiency in legumes. Legumes use more soil N (such as ${\mathrm{NO}}_{3}^{–}$ and ${\mathrm{NH}}_{4}^{+}$) as the main nitrogen source rather than through SNF. There is also a malate accumulation in both types of nodules and more ureides export in determine nodules under Pi deficiency. A red or purple label means the metabolite content or enzyme activity is up- or down-regulated, respectively. Arrows up (↑) and down (↓) mean the nutrient uptake is up- or down-regulated, respectively. SNF, symbiotic nitrogen fixation; PEP, phosphoenolpyruvate; PEPC, phosphoenolpyruvate carboxylase; OAA, oxaloacetate; MDH, malate dehydrogenase; ASN, asparagine.

According to previous studies, under low Pi availability, nodules would largely decrease the utilization of atmospheric nitrogen as the nitrogen source and utilize more soil nitrogen (such as ${\mathrm{NO}}_{3}^{–}$ and ${\mathrm{NH}}_{4}^{+}$) instead, as shown in Figure 2 (Valentine et al., 2017). The reason for such a shift is not only that ${\mathrm{NO}}_{3}^{–}$ itself is a strong inhibitor of nodulation and SNF, but also that the consumption of carbon as an energy source for nitrogen uptake from soil is less than that for SNF (Minchin and Witty, 2005). In *Virgilia divaricata*, the carbon cost related to SNF ranges from 3.3 to 6.6 gCg^-1^-N (gram of carbon was used when fixed each gram of N_2_), while the carbon cost of ${\mathrm{NO}}_{3}^{–}$ reduction is not more than 2.5 gCg^-1^-N. Hence, the assimilation of nitrogen from the soil would save more organic carbon for plant growth (Minchin and Witty, 2005). In SNF nodules, the host plant provides photosynthetically fixed carbon to symbiosomes as the major energy source. In turn, bacteria in the nodule fix N_2_ by the activities of nitrogenases and release ${\mathrm{NH}}_{4}^{+}$ to the host cells. The carbon transported to symbiosomes is mainly in the form of TCA cycle dicarboxylates (such as malate) and is also used as the carbon skeletons for the syntheses of amino acids from N_2_ (Rosendahl et al., 1990; Valentine et al., 2017; Figure 2). For example, in *L. angustifolius* cv. Wonga, there is an increased rate of malate synthesis via PEPC and MDH under long-term Pi deficiency. Although malate is the major energy source for symbiosomes, high malate accumulation could inhibit N_2_ fixation and nitrogen assimilation (Le Roux et al., 2008).

#### Nitrogen Export From Nodules

As aforementioned, in exchange for the nutrient supply, ammonia and/or alanine and Asp are translocated into the legume cytosol from the symbiosome and then assimilated into ureides or amides. Under Pi deprivation, legumes prefer exporting more ureides, due to the lower carbon cost (Figure 2). This was demonstrated through an experimental determination of C and N budgets in the tropical legume cowpea and the temperate legume lupin. The results were 1.4 gCg^-1^ fixed N in cowpea which forms ureide-exporting nodules, and a minimum of 3.9 gCg^-1^ fixed N in lupin which forms amide-exporting nodules. The result indicates that the export of ureides is more economical in the use of carbon (Atkins, 1991). The shift to higher amounts of ureide export under Pi stress was also observed in soybean, where there was higher accumulation of ureides relative to amino acids in nodules (Le Roux et al., 2009). The switch to ureide exports may reduce the carbon cost under Pi stress, which has a positive effect on legume growth.

#### Pi Homeostasis in Legume Nodules

Under Pi stress, legumes exhibit very flexible mechanisms to maintain Pi homeostasis in nodules. On the one hand, legumes increase Pi acquisition from the external environment. On the other hand, plants adopt flexible Pi recycling and internal Pi conservation mechanisms to improve Pi remobilization (Nasr Esfahani et al., 2016; Saad and Lam-Son, 2017).

##### Pi acquisition from the external environment

Pi acquisition from the external environment by plants is affected by nitrogen metabolism. Pretreatment with ${\mathrm{NH}}_{4}^{+}$ and ${\mathrm{NO}}_{3}^{–}$ can increase Pi uptake from soil in *Zea mays* roots (Smith and Jackson, 1987). Acquisition of ${\mathrm{NH}}_{4}^{+}$ can lead to a release of proton (H^+^) that decreases the pH in the rhizosphere, which in turn stimulates Pi solubility and uptake (Zhao et al., 2009). Legumes tend to take up ${\mathrm{NH}}_{4}^{+}$ and ${\mathrm{NO}}_{3}^{–}$ from the soil through the roots in both Pi stress and Pi sufficient conditions (Dan and Brix, 2009). In turn, more acquisition of ${\mathrm{NH}}_{4}^{+}$ can improve the availability of Pi in the external environment.

Plant acid phosphatases (APases) can hydrolyze organic phosphates to improve soil Pi availability in legumes (Figure 2). Under Pi stress, APases become catalytically better using the same amount of nodule crude extract, which is an efficient way to utilize organic phosphates (Araújo et al., 2008). In addition, phytases can hydrolyze phytate into myoinositol and Pi to improve the Pi level in the rhizosphere for uptake through the roots (Araújo et al., 2008). The expression and activities of phytases are also induced by Pi deprivation in *P. vulgaris* nodules. Two recombinant inbred lines (RILs) of *P. vulgaris*, RILs 115 (P-efficient) and 147 (P-inefficient), were inoculated with *R. tropici* CIAT 899 strain and planted in Pi-deficient conditions. Under low Pi condition, the accumulation of phytase transcripts was observed in the nodules of both lines, and it is more enhanced in Line RIL115 than in Line RIL147. The increase in phytase and phosphatase enzyme activities and higher SNF efficiency were found in RIL115 under Pi-deficient treatment, indicating a possible role of phytase activity in nodules to maintain SNF under Pi deficiency condition (Lazali et al., 2013).

The uptake of Pi is also under the regulation of high-affinity Pi transporters. Plants have two identified Pi uptake mechanisms: a high-affinity system that may be up-regulated under low Pi condition and a low-affinity system which is constitutively expressed (Hernandez et al., 2009; Nasr Esfahani et al., 2016). In this review, we focus on the high-affinity Pi transporters. Most high-affinity Pi transporters are induced by Pi starvation and expressed in the root hairs and root epidermis which are directly exposed to soil Pi (Liu et al., 2008). Among Pi transporters, the Pht1 family is the most intensively studied in plants (Gu et al., 2016). Pht1, localized in the plasma membrane, is directly related to Pi uptake from soil and Pi translocation in the plant. Some Pht1s are directly under the control of the central regulators of Pi starvation signaling, the phosphate starvation response (PHR) transcription factors (Rubio et al., 2001; reviewed in Gu et al., 2016). In *G. max*, 15 *Pht1* paralogs have been identified by bioinformatics and experimentation. The expressions of all 15 *GmPht1*s are up-regulated under low Pi condition compared to high Pi condition. By overexpressing each *GmPHT1* paralog in the yeast Pi-transporter mutant PAM2 (*Δpho84 Δpho89*), *GmPht1;1, GmPht1;2, GmPht1;5, GmPht1;7*, and *GmPht1;10* are found to be high-affinity Pi transporters and the others are lower affinity Pi transporters (Fan et al., 2013). A Pi starvation-induced high-affinity Pi transporter, *GmPT5* (*Glyma10g04230*), was also identified, which was considered to play an important role in maintaining nodular Pi homeostasis. *GmPT5* is expressed in the junction area between roots and young nodules, and mainly functions in transporting Pi from roots to nodules. Higher expression of *GmPT5* leads to more Pi being transported from root to nodule, which is beneficial for maintaining SNF (Qin et al., 2012).

##### Internal phosphate recycling and phosphate conservation

Besides the uptake of Pi from the external environment, legume nodules also develop phosphate recycling and phosphate conservation mechanisms under Pi deficiency (Figure 2) (Saad and Lam-Son, 2017). A report showed that chickpea could re-allocate Pi from roots to nodules when facing Pi stress. A sharp reduction in Pi (by around 78%) in roots was detected in chickpea, which the researchers assumed that the missing Pi was transported into nodules in order to prevent the complete depletion of nodular Pi (Nasr Esfahani et al., 2016). Common bean was also reported to mobilize Pi from nucleic acids and phospholipids (Hernandez et al., 2009). Under low Pi conditions, *V. divaricata* exhibited a lower Pi uptake rate but higher levels of phosphohydrolase exudation. This suggests that *V. divaricata* may prefer to recycle internal nodular Pi pools and use alternate bypass routes to conserve Pi rather than directly uptaking Pi from soil (Vardien et al., 2016).

### Regulation of Metabolism in Legume Nodules of Host Plants

#### Regulators of Carbon and Nitrogen Metabolism

It is an important challenge for legumes to maintain a balance between supplying the nodules with the amount of carbon required for nitrogen fixation while retaining sufficient carbon for growth, as well as keeping the nitrogen efflux from the nodules at an optimum level. The host plant regulates the symbiotic process by controlling nodule development and nodule numbers as well as by adjusting the nodule turnover and the level of nitrogen fixation (Ferguson et al., 2010; Sulieman and Schulze, 2010). The balance between plant nitrogen demand and nitrogen fixation rate is finely tuned to reach the nitrogen concentration that allows maximum plant growth. In common bean, nitrogen translocated from senescing lower leaves to nodules resulted in lower nitrogen fixation rate (Fischinger et al., 2006). Autoregulation of nodulation (AON) is the main systemic negative feedback mechanism of host plants to negatively regulate SNF in nodules (Ferguson et al., 2010). AON has been identified using several legume mutants with the super nodulation phenotype, such as the *G. max* mutant *nts-1, M. truncatula* mutant *sunn*, and *L. japonicus* mutant *har1*, were found to lack in a leucine-rich repeat receptor-like protein kinase for AON in shoots (Krusell et al., 2002; Nishimura et al., 2002; Schnabel et al., 2005). CLAVATA3/embryo-surrounding region (CLE) peptides are a group of small (12–13 amino acids) secreted peptides derived from the C-terminal region of preproproteins. In *M. truncatula*, it has been suggested that *MtCLE12* or *MtCLE13* plays a role for CLE signaling in controlling nodule numbers. These peptides are generated in roots and transported to leaves via the xylem to trigger the AON response (Mortier et al., 2010). *LjCLE-RS1* and *LjCLE-RS2* were also found to be involved in regulating nodule formation in *L. japonicus* (Magori and Kawaguch, 2010).

#### Regulator for Pi Homeostasis

Legumes have evolved to adopt strategies to maintain the nodular Pi homeostasis (Sulieman and Tran, 2015). Multiple genes and proteins work as regulators (Rubio et al., 2001; Yao Z.F. et al., 2014; Sulieman and Tran, 2015). A MYB-CC-type transcription factor (*PHR1*), a small non-coding RNA (microRNA399), and proteins containing the SYG1/PHO81/XPR1 (SPX) domain all play important roles in Pi stability in nodules, and are considered to be vital regulators (Xue et al., 2017). DNA methylation may also be involved in the nodular Pi homeostasis regulation, but it still needs further research (Kim et al., 2015; Yong-Villalobos et al., 2015; Crampton et al., 2016).

In soybean, 35 GmPHR members have been identified. Among them, *GmPHR25* is induced by Pi starvation and it in turn increased the transcripts of 11 out of 14 high-affinity Pi transporters as well as those of 5 other Pi starvation-responsive genes (Xue et al., 2017). This evidence indicated that *GmPHR25* is a vital Pi homeostasis regulator in soybean (Xue et al., 2017). Besides small non-coding RNAs, long non-coding RNAs (lncRNAs) are also involved in Pi homeostasis. In the legume model plant, *M. truncatula*, three phosphate deficiency-induced LncRNAs (*PDILs*) have been characterized through their corresponding *Tnt1* mutants. *PDIL1* suppresses the degradation of *MtPHO2*, working as a positive Pi homeostasis regulator. However, *PDIL2* and *PDIL3* may directly inhibit Pi transporters, therefore working as negative regulators (Wang et al., 2017).

Proteins containing the *SPX* domain are considered to be vital regulators in the plant Pi signaling network (Yao Z.F. et al., 2014). In common bean, *PvSPX1–PvSPX3* are all induced by Pi starvation, but *PvSPX1* had higher sensitivity and faster response to Pi starvation than the other two *PvSPX*s. Ten Pi starvation-responsive genes are induced by overexpressing *PvSPX1*, even with an increase in Pi concentration at the same time. It was further shown that the overexpression of *PvPHR1* resulted in a decrease in *PvSPX1* transcripts. Therefore, *PvSPX1* is a positive regulator in maintaining Pi homeostasis and is itself regulated by *PvPHR1* (Yao Z.F. et al., 2014). But in soybean, *GmSPX1* is a negative regulator in the Pi signaling network, and may be involved in phosphate starvation by inhibiting the expression of *GmMYB48*, a phosphate starvation-induced gene (Zhang et al., 2016). *GmSPX3* is also induced by Pi starvation, and considered to be a positive regulator which induces seven Pi starvation-responsive genes in soybean hairy root (Yao Z. et al., 2014). Genes found to be associated with legume Pi homeostasis are listed in Table 2.

**Table 2:** Genes involved in legume Pi homeostasis.

Gene	Species	Annotation	Response to Pi deficiency	Description	References
*PvPHR1*	*Phaseolus vulgaris*	MYB-CC TF	Induced	Overexpression of *PvPHR1* can activate a subset of Pi starvation-responsive genes and increase Pi concentration.	Valdés-López et al., 2008; Moris et al., 2004
*GmPHR25*	*Glycine max*	MYB-CC TF	Induced	Overexpression of GmPHR25 can activate 11 high-affinity Pi transporters and 5 Pi starvation-responsive genes and increase Pi concentration.	Xue et al., 2017
*GmSPX1*	*Glycine max*	SPX domain-containing protein	Induced	Induced by Pi starvation by interacting with *GmMYB48*; Overexpression of *GmSPX1* can decrease Pi concentration.	Zhang et al., 2016
*GmSPX3*	*Glycine max*	SPX domain-containing protein	Induced	Overexpression of *GmSPX3* can activate 7 Pi starvation-responsive genes and increase Pi concentration.	Yao Z. et al., 2014
*PvSPX1*	*Phaseolus vulgaris*	SPX domain-containing protein	Induced	Overexpression of *PvSPX1* can activate 10 Pi starvation-responsive genes and increase Pi concentration.	Yao Z.F. et al., 2014
*PvPHO2*	*Phaseolus vulgaris*	Ubiquitin E2 conjugase	Suppressed	Regulate Pi starvation responses	Valdés-López and Hernández, 2008
*CaPHT1;4*	*Cicer arietinum*	High-affinity Pi transporter	Induced	Involved in Pi acquisition and mobilization	Nasr Esfahani et al., 2016
*GmPT5*	*Glycine max*	High-affinity Pi transporter	Induced	Involved in Pi transportation from root to nodule	Qin et al., 2012
*CaPHO1*	*Cicer arietinum*	Phosphatase 1	Unchanged	Involved in Pi transporting into the xylem	Nasr Esfahani et al., 2016
*LjPHO3*	*Lotus japonicus*	Sucrose/H+ symporter	NA	Mediate Pi starvation responses	Qin et al., 2016
*PvTIFY*	*Phaseolus vulgaris*	JAZ TF	Induced	Involved in the regulation of Pi deficiency	Aparicio-Fabre et al., 2013
*MtmiR399*	*Medicago truncatula*	MicroRNA	Induced	Regulate *PHO2* and Pi transporter	Li et al., 2018
*MtPDIL1*	*Medicago truncatula*	LncRNA	Induced	Suppress degradation of *MtPHO2*	Wang et al., 2017
*MtPDIL2*	*Medicago truncatula*	LncRNA	Suppressed	Suppress Pi transporter	Wang et al., 2017
*MtPDIL3*	*Medicago truncatula*	LncRNA	Suppressed	Suppress Pi transporter	Wang et al., 2017

## Metabolism and Transport in Bacteroids

### Nitrogen Assimilation

#### Nitrogenase Complex

Symbiotic nitrogen fixation by rhizobia in legume root nodules is carried out through the nitrogenase enzyme complex. Nitrogenase catalyzes the following reaction:

$${\text{N}}_{\text{2}}{\text{+8e}}^{\text{-}}{\text{+8H}}^{\text{+}}\text{+16ATP}\to {\text{2NH}}_{\text{3}}{\text{+H}}_{\text{2}}\text{+16ADP+16Pi}.$$

This reaction describes the reduction of nitrogen to ammonia, and is associated with a high energetic cost (ATP). The enzymatic complex of nitrogenase consists of two enzymes: dinitrogenase reductase, a dimeric Fe-protein encoded by the *nifH* gene, and dinitrogenase, a tetrameric FeMo-protein encoded by the *nifDK* gene (Rascio and La Rocca, 2013; Rubio and Ludden, 2015). These enzymes are rapidly deactivated in the presence of atmospheric concentrations of oxygen (Dixon and Wheeler, 1986).

#### Regulation of Nitrogenase Activity

Nitrogenase activity is reduced at high oxygen concentrations. Nodules provide low oxygen environments for the N_2_-fixing bacteroids, as observed in soybean nodules where the oxygen concentration is 56 nM (Kuzma et al., 1993) and even lower concentrations have been registrated (Appleby, 1984). Therefore, oxygen tension is a key regulator of genes required for N_2_ fixation, nitrogenase synthesis (*nif* genes), and microoxic respiration (*fix* genes). The nitrogen fixation signaling pathway differs between rhizobium strains. In *S. meliloti*, the heme-containing FixL, a signal-transducing membrane protein, acts as the oxygen sensor for the system. In the absence of oxygen, FixL autophosphorylates and transfers the phosphate to FixJ (Gilles-Gonzalez et al., 1991; Lois et al., 1993). This transcriptional factor in turn regulates positively the expression of two regulatory genes, *nifA* and *fixK*. NifA is a transcriptional activator that controls the expressions of nitrogen fixation genes, *fixABCX, nifN, nifB*, and *nifHDK* (Gong et al., 2006). FixK upregulates the expression of *fixNOQP* and *fixT* (Batut et al., 1989; Foussard et al., 1997). FixT represses the expression of *nifA* (Foussard et al., 1997) and FixNOQP is a cytochrome terminal oxidase *cbb3* with a high affinity for oxygen that allows ATP production required for the nitrogen fixation process (Preisig et al., 1993). However, in *B. japonicum*, the FixLJ system is not required to induce the expression of *nifA* (Fischer and Hennecke, 1987), but instead it is RegSR that mediates positive control to transcription of *nifA* (Lindemann et al., 2007).

FixLJ cascade system varies in some bacteria. FnrN, belonging to the CRP-Fnr family of global transcriptional factors found in bacteria, acts as the oxygen sensor in *R. leguminosarum* bv. *viciae* VF39 and *Rhizobium etli* CNPAF512 (Patschkowski et al., 1996; Moris et al., 2004). Both organisms synthesize FnrN and FixK. In *R. leguminosarum*, FnrN is the main oxygen sensor responsible for the induction of *fixNOQP* and is in turn induced by the FixL protein (Boesten and Priefer, 2004). In contrast, the expression of *fnrN* is autoregulated in *R. etli* CNPAF512 (Moris et al., 2004). Other cases of rhizobia oxygen-response cascade have been previously described in detail (Terpolilli et al., 2012).

### Dicarboxylate Metabolism

Most rhizobia are obligate aerobes. The C_4_-dicarboxylic acids supplied by the host plant must be metabolized through the tricarboxylic acid (TCA) cycle in the bacteroid. Malate, the primary carbon source of bacteroids, is converted to pyruvate and CO_2_ via the NAD^+^-dependent malic enzyme (DME). Pyruvate is subsequently decarboxylated by pyruvate dehydrogenase to form acetyl-coenzyme A (acetyl-CoA) and enters the TCA cycle. DME is required to provide pyruvate to the TCA cycle in *S. meliloti* and *Azorhizobium caulinodans* (Zhang Y. et al., 2012). *S. meliloti* possesses two distinct malic enzymes, an NAD(P)^+^-dependent enzyme (DME) (EC 1.1.1.39) and a strictly NADP^+^-dependent enzyme (TME) (EC 1.1.1.40) (Driscoll and Finan, 1993, 1996; Voegele et al., 1999). In addition, acetyl-CoA can be produced alternatively by phosphoenolpyruvate carboxykinase (PCK) which catalyzes the decarboxylation of OAA to PEP. Symbiotic phenotypes of *pck* mutants vary depending on the host plant. *pckA* mutants of *R. leguminosarum* MNF3085 fix nitrogen at rates comparable to wild type, while *pckA* mutants of *S. meliloti* show a reduced N_2_ fixation capacity (McKay et al., 1985; Finan et al., 1991). In *R. leguminosarum* and *S. fredii* strain NGR234, DME or a pathway involving PCK and pyruvate kinase (PYK) can synthesize the precursors required for SNF (Zhang Y. et al., 2012). Enzymes participating in the TCA cycle have been identified in *B. japonicum* strain USDA110 (Sarma and Emerich, 2006), *R. leguminosarum* (McKay et al., 1985), *S. meliloti* (Djordjevic, 2004), and *R. tropici* (Romanov et al., 1994). However, TCA cycle involvement probably varies among rhizobia. Evidence shows that *S. meliloti, R. tropici*, and *R. leguminosarum* utilize the full oxidative TCA cycle to provide ATP, precursors of amino acid synthesis, as well as reducing equivalents for N_2_ fixation. Enzymes of the TCA cycle appear to be essential for nitrogen fixation in *S. meliloti* since mutations in succinate dehydrogenase (*sdh*), malate dehydrogenase (*mdh*), isocitrase dehydrogenase (*icd*), 2-oxoglutarate dehydrogenase, aconitase (*acnA*), and citrate synthase (*gltA*) abolish N_2_ fixation, despite that nodules were formed (Duncan and Fraenkel, 1979; McDermott and Kahn, 1992; Mortimer et al., 1999; Dymov et al., 2004; Koziol et al., 2009). In contrast, *B. japonicum* shows a higher metabolic plasticity. Mutations in fumarase (*fumC*), ICDH (*idhA*), alpha-ketoglutarate dehydrogenase (*agdA*), and acotinase (*acnA*) in *B. japonicum* USDA110 still show phenotypes capable of fixing nitrogen (Acuña et al., 1991; Thöny-Meyer and Künzler, 1996; Green and Emerich, 1997; Shah and Emerich, 2006). Transcriptome analyses of *R. etli* bacteroids have suggested that the TCA cycle is inactive in these bacteroids (Vercruysse et al., 2011), but additional experiments are required to explain how the bacteroids obtain the ATP necessary for nitrogen fixation in these cases. A comparative metabolic profiling study between free-living *R. leguminosarum* and pea bacteroids showed that the TCA cycle is not the only path to oxidize dicarboxylic acids derived from the host plant in the bacteroids (Terpolilli et al., 2016). Metabolic profiling and flux analysis revealed that pea bacteroids divert acetyl-CoA into TCA and the production of lipid or polyhydroxybutyrate (PHB). These findings suggest new pathways for electron allocation in nitrogen-fixing bacteroids where lipogenesis may be a requirement in legume nodules.

### Carbon Storage

The legume–rhizobium symbiosis determines the accumulation of specific metabolites inside the microsymbiont. Excess carbon and reducing power provided by the host plant can be stored as polymers, glycogen, or lipids in bacteroids (Figure 1). PHB is accumulated in large cytoplasmic granules in the bacteroids that form determinate nodules (such as in common bean and soybean), but not in the indeterminate nodules of alfalfa, garden pea, and chickpea (Table 1; Tombolini and Nuti, 1989; Kim et al., 1996).

The PHB biosynthesis pathway consists of three steps. β-Ketothiolase (PhaA) catalyzes the first step in this pathway with the formation of acetoacetyl-CoA from acetyl-CoA. Acetoacetyl-CoA is then reduced to D-β-hydroxybutyryl-CoA by an NADH-dependent acetoacetyl-CoA (PhaB), and then PHB synthase (PhaC) catalyzes the final formation of PHB (Lodwig and Poole, 2003). In *S. meliloti* and *R. etli, phaA* and *phaB* form an operon, *phaAB*.

Fix^+^ symbiotic phenotypes have been observed in *S. meliloti* when mutations were introduced to disable the synthesis of PHB or alter the ability to utilize PHB cycle intermediates to support growth (Povolo et al., 1994; Aneja and Charles, 1999; Cai et al., 2000). Effective nodules were also observed when *R. leguminosarum* mutant strains defective in the *phaC* gene were used to inoculate bean (determinate nodules) or pea (indeterminate nodules) (Lodwig et al., 2005). However, a reduced nitrogen fixation capability was observed when PHB synthesis was abolished in *A. caulinodans*. In *A. caulinodans*, impaired PHB synthetase activity results in the loss of nitrogen fixation capacity both *ex planta* and in symbiosis with the tropical legume *Sesbania rostrata* (Mandon et al., 1998). These evidences suggest that PHB may play certain roles in nitrogen fixation of some legume–rhizobium systems.

Polyhydroxybutyrate synthesis is regulated in *R. etli* and *S. meliloti* by the PHA (polyhydroxyalkanoate) regulator (PhaR), previously called AniA (Povolo and Casella, 2000; Encarnación et al., 2002). PhaR homologs in other bacterial species bind to their own promoters and to the promoter of *phaP* (Maehara et al., 2001). PhaP, a phasin, binds to the surface of PHB granules and can control the size of the PHB granules (Kuchta et al., 2007; Mezzina et al., 2014). In *Bradyrhizobium diazoefficiens*, the regulation of PHB synthesis is mediated through the interaction between PhaR and FixK_2_, regulators associated with the synthesis of PHB and microoxic metabolism (Quelas et al., 2016).

Glycogen is co-produced with PHB in free-living rhizobia such as *R. leguminosarum* and *S. meliloti* under nutrient-limiting conditions (Povolo et al., 1994; Lodwig et al., 2005). The role of glycogen in SNF has been studied in some rhizobia. A glycogen synthase mutant (*glgA*) of *R. tropici* showed an enhanced symbiotic performance as measured by an increase in plant dry weight and nodule number, but the mechanisms behind this phenotype are uncertain (Marroquí et al., 2001). In garden pea bacteroids, a *glgA* mutant of *R. leguminosarum* did not alter nitrogen fixation rates, as indicated by the acetylene reduction assay for nitrogenase activity. Plants inoculated with a *phaC*/*glgA* double mutant resulted in similar phenotypes to wild-type-inoculated plants (Lodwig et al., 2005). In contrast, the *glgA* mutant of *S. meliloti* resulted in lower levels of N_2_ fixation in both *M. truncatula* and *M. sativa* (Wang et al., 2007). Thus, the SNF capability of *S. meliloti* is affected by both PHB and glycogen availability.

In *Bradyrhizobium* sp. ORS278, studies have shown that mutations in genes involved in the Calvin–Benson–Bassham (CBB) cycle result in deficiency in nitrogen fixation. The CBB cycle consumes 3 mol of ATP and 2 mol of reducing equivalents per mol of carbon dioxide. The symbiotic phenotypes suggest that *Bradyrhizobium* sp. ORS278 uses the Calvin cycle as a reductant store to regulate carbon flux. Specifically, phosphoglycerate kinase (CbbK) and ribulose 1,5-biphosphate carboxylase (RuBisCO) large chain (CbbL1) have been identified as being involved in the symbiotic process (Bonaldi et al., 2010; Gourion et al., 2011). Thus, the relevance of different carbon storage compounds in the symbiotic process varies depending on the rhizobial strain.

### Transport in Bacteroids

#### Symbiotic Membrane: A Regulatory Barrier

Mature bacteroids drive nitrogen fixation inside root nodules, releasing ammonia to the plant cell cytosol to be incorporated into nitrogenous compounds. Ammonia assimilation is severely reduced in bacteroids due to a coordinated response where growth-associated pathways are switched off and biosynthesis of amino acids is downregulated but still present (Li et al., 2013). However, bacteroids require the supply of some plant-derived amino acids to support bacteroid development (Prell et al., 2009; Mulley et al., 2011; reviewed in Dunn, 2014). The supply of amino acids is plant-type specific (Randhawa and Hassani, 2002). For example, alfalfa is not able to provide sufficient histidine to the histidine auxotrophs of *S. meliloti* in nodules (Malek and Kowalski, 1977) unlike the cowpea host plant where similar mutants of rhizobial strain IRC256 are able to fix nitrogen (McLaughlin et al., 1987). The transport of nutrients is regulated by the symbiosome membrane (SM). SM is a plant-derived membrane that controls the metabolite exchanges in the legume–rhizobia symbiosis via specific transport systems and encloses a single bacteroid in indeterminate nodules or several bacteroids in determinate nodules (Clarke et al., 2014). Different transport mechanisms have been identified across the legume SM such as carbon, nitrogen, and cation transport systems, but most of them are not characterized (Day et al., 2001; Clarke et al., 2015). The main metabolite transport systems are schematically represented in Figure 1.

#### Dicarboxylate Transport

C_4_-Dicarboxylates are the major metabolites that are transported across SM to generate ATP in the bacteroids. A dicarboxylate transporter for malate and succinate has been identified in the soybean SM (Udvardi et al., 1988). This transporter possesses a higher affinity for malate than for succinate. However, the gene encoding the transporter has not been identified in any legume. A dicarboxylate transporter has been characterized in the SM of a non-legume, *Alnus glutinosa* AgDCAT1 was shown to transport dicarboxylates (malate, succinate, fumarate, and OAA) when expressed in *Escherichia coli* (Jeong, 2004). AgDCAT1 belongs to the peptide transporter family (PTR) and proteins from this family are candidates for dicarboxylate transporters in legumes since experimental evidence shows that PTR-encoding genes are induced in legume nodules (Colebatch et al., 2004; Libault et al., 2010). Members of this family have been identified in the proteomic studies of the soybean SM, but their functions have not been characterized (Clarke et al., 2015). In rhizobia, a C_4_-dicarboxylate transport system (Dct) has been identified and characterized. Dct consists of a permease encoded by *dctA* and a two-component sensor-regulator system (DctBD) encoded by *dctB* and *dctD*. DctBD responds to the presence of C_4_-dicarboxylates and regulates *dctA* expression (reviewed in Yurgel and Kahn, 2004). *dctA* mutants of *Rhizobium trifolii* are unable to transport dicarboxylates and form ineffective nodules (Fix*^-^*) in *Trifolium repens* and *Trifolium pratense*. The resulting infected plant cells accumulated large amounts of starch (Ronson et al., 1981).

#### Ammonia and Ammonium Transport

The ammonia produced by nitrogen fixation in bacteroids is probably protonated to ammonium after diffusing into the peribacteroid space (PBS) or symbiosome space (SS, the region between the SM and the bacteroid) (Figure 1). An H^+^-ATPase pumps H^+^ into the PBS, generating an acidic environment (Udvardi et al., 1991) and forming a membrane potential (Udvardi and Day, 1989). ATPase activity has been detected on the SM of soybean (Blumwald et al., 1985; Fedorova E. et al., 1999), lupin (Robertson et al., 1978; Domigan et al., 1988), and garden pea root nodules (Szafran and Haaker, 1995). Re-uptake of ${\mathrm{NH}}_{4}^{+}$ by bacteroids is prevented by the repression of the bacteroid ammonium carrier, Amt, during the symbiotic state (Howitt et al., 1986). At present, two pathways have been proposed for the transport of fixed nitrogen into the host plant: a monovalent cation channel for ${\mathrm{NH}}_{4}^{+}$ (Whitehead et al., 2008) and an aquaglyceroporin, nodulin 26 (Nod26), for NH_3_ transport. Nod26 is a transmembrane protein able to transport H_2_O, NH_3_, and other solutes (Weaver et al., 1994; Rivers et al., 1997; Hwang et al., 2010). It was first identified in the soybean SM (Fortin et al., 1987) and constitutes the major protein component of the SM (Rivers et al., 1997).

#### Amino Acid Transport

Host plants supply amino acids to the microsymbionts during the differentiation of rhizobia to bacteroids and for nitrogen fixation (Mulley et al., 2011). Recent analyses of the soybean proteome have revealed a putative amino acid transporter (GmAPC1), a homolog to members of the amino acid-polyamine-organocation (APC) family of transport proteins (Clarke et al., 2015). Previous studies have shown that the soybean SM is permeable to alanine and Asp (Whitehead et al., 1998). An Asp transporter has been identified in SM vesicles from root nodules of garden pea (Rudbeck et al., 1999).

In addition, rhizobia also encode transport systems that regulate the exchange of amino acids between the bacteroids and the host cell (Udvardi and Poole, 2013). For instance, while the bacteroids formed by *R. leguminosarum* bv. *viciae* in the indeterminate pea nodules are auxotrophs for branched amino acids, branch-chain amino acid transporters, Aap (AapJQMP) and Bra (BraDEFGC) are developed in the bacteroids (Prell et al., 2009). This symbiotic phenotype is known as symbiotic auxotrophy (Dunn, 2014) and occurs in both determinate and indeterminate nodules (de las Nieves Peltzer et al., 2008; Prell et al., 2010; di Cenzo et al., 2015).

### Metabolic Modeling of SNF

Constraint-based modeling enables the determination of the metabolic capabilities of an organism (Feist et al., 2009; Oberhardt et al., 2009). These metabolic models have been used successfully to bridge the gap between current knowledge and metabolic phenotypes (Schellenberger et al., 2011). Metabolic reconstructions are based on physiological and biochemical information on primary and specialized metabolic pathways, genome information, and available -omics data. These models have allowed for the prediction of the metabolic behavior of microorganisms under different nutrient availabilities and simulated gene deletions or over-expressions (Contador et al., 2015; Razmilic et al., 2018). In an attempt to get new insights into the genetic interactions that orchestrate the complex metabolic interactions in nitrogen-fixing bacteria, metabolic models have been used to study the rhizobial metabolism. Genome-scale models have been combined with experimental data to identify essential genes and to simulate the metabolic behaviors of rhizobia under nitrogen fixation conditions in legume nodules. High-throughput experimental data aimed to identify key genes, proteins and metabolites have been used in these studies to validate the model predictions.

At present, few manually curated rhizobial reconstructions are available despite their important roles in sustainable agriculture. These reconstructions cover different metabolic tasks from only the symbiotic process that takes place in the rhizobium to the complete set of metabolic reactions of the bacteria cell. *i*OR363, a reconstruction for *R. etli* CFN42, was the first model available for any rhizobium (Resendis-Antonio et al., 2007). This model describes the SNF process inside the determinate nodules of *P. vulgaris* and includes the main reactions associated with the nitrogen fixation process and the metabolic pathways, such as PHB synthesis, that allow simulating the accumulation of this polymer during the symbiotic process (Resendis-Antonio et al., 2007). Also, a symbiotic reaction was defined to represent this specific legume–rhizobia symbiosis. The symbiotic reaction describes the exchange of nutrients between the host plant and the bacteroid to establish symbiotic relationships and to obtain or produce chemical compounds required by the process of nitrogen fixation. A symbiotic auxotrophy hypothesis and nitrogenase requirements were included in its definition. This model has been updated to include new data sets available of this system (Resendis-Antonio et al., 2011, 2012).

On the other hand, the metabolic reconstructions for *S. meliloti* have different scopes. *i*HZ565 was designed to represent the SNF of *S. meliloti* 1021 with host plants such as alfalfa (Zhao et al., 2012). The reconstruction process gave insight about the missing context-specific information and genome annotation errors. Additionally, a new symbiotic reaction was proposed to capture specific SNF mechanisms of *S. meliloti* in indeterminate nodules. The first representation of the whole metabolism of a rhizobium cell was built for *S. meliloti* (*i*GD1575) to enable the characterization of the metabolic capabilities of rhizobia in bulk soil, rhizosphere, and nodule (di Cenzo et al., 2016). *i*GD1575 encompasses 94% of the genes present in *i*HZ565. Recently, *i*GD1575 and gene essentiality experiments were used to build a model of the core metabolism of *S. meliloti* (di Cenzo et al., 2018). Pfau et al. (2018) have described the symbiotic relationship of *M. truncatula* with *S. meliloti* through constraint-based modeling (Pfau et al., 2018). Metabolic models were used to study the metabolic exchange between the host plant and the rhizobial symbiont. An *in silico* representation of the metabolic network of *B. diazoefficiens* USDA110 (*i*YY1101) has also been constructed (Yang et al., 2017). This reconstruction was used to build context-specific models to describe the metabolic differences observed between the free-living state of *B. diazoefficiens* USDA110 and symbiotic bacteroid. These models will provide a starting point for building metabolic reconstructions of closely related organisms such as other rhizobial strains and modeling frameworks to study these symbiotic interactions (Baumler et al., 2011; Monk et al., 2013; Ong et al., 2014). Table 3 summarizes the available metabolic models of rhizobia.

**Table 3:** Available metabolic reconstructions of rhizobia.

Model	Organism	Scope	Reference
*i*OR363	*R. etli*	SNF^a^	Resendis-Antonio et al., 2007
*i*OR450	*R. etli*	SNF^a^	Resendis-Antonio et al., 2012
*i*HZ565	*S. meliloti*	SNF^a^	Zhao et al., 2012
*i*GD1575	*S. meliloti*	Whole cell	di Cenzo et al., 2016
*i*GD726	*S. meliloti*	Core metabolism	di Cenzo et al., 2018
*i*YY1101	*B. diazoefficiens*	Whole cell	Yang et al., 2017

*^*a*^SNF, symbiotic nitrogen fixation.*

## Conclusion

The regulatory mechanisms regarding the formation and maintenance of root nodules for SNF are multi-faceted and association-specific, but they all involve the tight regulation of the flow of metabolites between the host plants and the bacteroids, as well as the different levels of controls over each step in the metabolic pathways through the metabolic enzymes involved and the transcription factors that regulate their expressions. Each host plant–rhizobium association has its own unique requirements and control mechanisms despite the overall common features. It is therefore critical that we have as much understanding as possible of the detailed mechanisms that make up this symbiotic relationship.

## Author Contributions

H-ML designed the conceptual framework of this paper and coordinated the writing. AL and KF wrote the part on metabolism and regulation of host plant. CC wrote the part on metabolism and regulation of bacteroids and metabolic models. AL, CC, and H-ML revised and polished the article.

## Conflict of Interest Statement

The authors declare that the research was conducted in the absence of any commercial or financial relationships that could be construed as a potential conflict of interest.
